# Combined transcranial photobiomodulation and neurofeedback in PANS/PANDAS: a retrospective case series

**DOI:** 10.3389/fpsyt.2026.1865423

**Published:** 2026-09-03

**Authors:** Brooke Hill

**Affiliations:** 1 Neuronic, Austin, TX, United States; 2 Butmeyer, Austin, TX, United States

**Keywords:** cognitive function, neurofeedback, neuroinflammation, neuromodulation, PANDAS, PANS, pediatric neuropsychiatry, transcranial photobiomodulation

## Abstract

**Background:**

PANS and PANDAS are neuroimmune conditions characterized by neuropsychiatric symptoms, including obsessive-compulsive features, cognitive dysfunction, and emotional dysregulation, that may persist despite multimodal treatment. Adjunctive neuromodulation approaches remain underexplored in this population.

**Methods:**

This retrospective observational case series describes two adolescents with persistent PANS/PANDAS symptoms despite more than 10 years of multidisciplinary care who received home-based transcranial photobiomodulation (tPBM) using a 1070 nm near-infrared device and adjunctive swLORETA neurofeedback as part of routine clinical care. Multimodal outcomes included the PANS 31-Item Symptom Rating Scale, Yale-Brown Obsessive Compulsive Scale, CogniFit CAB-Pro, BrainGauge, caregiver-reported functional outcomes, and qEEG.

**Results:**

Reductions in neuropsychiatric symptom burden and functional gains were observed during the observation windows. One participant showed a 73% reduction in PANS-31 severity with improvements across multiple cognitive domains; the other showed a 50% reduction in obsessive-compulsive symptom severity and progressed from homebound status to full school participation. Changes were observed across clinical, cognitive, functional, and qEEG measures; however, outcome timing differed across participants and instruments.

**Conclusion:**

These findings should be interpreted as preliminary, hypothesis-generating observations only. Because of the retrospective design, very small sample size, prior and concurrent interventions, and non-uniform assessment schedules, no conclusions regarding efficacy, causality, or attribution to either intervention or their combined use can be drawn. Prospective controlled studies are needed to evaluate efficacy, optimal sequencing, and intervention parameters.

## Introduction

PANS (pediatric acute-onset neuropsychiatric syndrome) and PANDAS (pediatric autoimmune neuropsychiatric disorders associated with streptococcal infections) are neuroimmune conditions characterized by neuropsychiatric symptoms, including obsessive-compulsive features, cognitive dysfunction, and emotional dysregulation (1, 2). These symptoms may persist despite multimodal treatment approaches, reflecting the clinical complexity of these conditions (2). The underlying pathophysiology is hypothesized to involve immune-mediated mechanisms affecting the basal ganglia and related cortico-striatal circuitry (1, 3), a network linking deep brain structures such as the caudate and putamen with frontal cortical regions involved in behavioral regulation.

Despite increasing recognition, management of PANS/PANDAS remains clinically challenging. Standard approaches may yield variable or incomplete responses, particularly in complex cases, and some patients continue to experience cognitive dysfunction, emotional dysregulation, and functional impairment despite multidisciplinary care (2). Consequently, there is growing interest in adjunctive interventions that target neural network regulation and neuroplasticity. However, evidence supporting such approaches in pediatric neuroimmune disorders remains limited, and their role within multidisciplinary care has not been established.

Transcranial photobiomodulation (tPBM) delivers near-infrared light to cortical tissue and has been investigated for potential effects on mitochondrial function, cerebral blood flow, and modulation of neuroinflammatory signaling (4–6). Although preliminary findings in several neurological and psychiatric conditions are encouraging, the available clinical evidence remains heterogeneous, with relatively few controlled studies and limited pediatric data.

Neurofeedback, including standardized low-resolution electromagnetic tomography (swLORETA) neurofeedback, has been investigated as a method for supporting self-regulation of distributed neural networks involved in attention, executive function, and emotional regulation (7–9). While some studies report improvements in selected neuropsychiatric conditions, systematic reviews have noted variability in protocols, outcome measures, and study quality, limiting firm conclusions regarding efficacy across disorders (8, 9).

Despite increasing interest in neuromodulation, published evidence specifically evaluating either tPBM or neurofeedback in PANS/PANDAS remains sparse. Most published literature has focused predominantly on immunological mechanisms, pharmacological management, and broader multidisciplinary care, with comparatively little attention directed toward interventions targeting functional brain networks.

Accordingly, this retrospective observational case series describes two adolescents with persistent PANS/PANDAS symptoms who received individualized tPBM and adjunctive swLORETA neurofeedback as components of routine multidisciplinary care. The objective was to describe observations across multiple clinical, cognitive, functional, and neurophysiological outcome domains and identify patterns that may generate testable hypotheses for future prospective investigation.

## Methods

### Study design

This retrospective observational case series describes two adolescents with persistent PANS/PANDAS symptoms who received home-based transcranial photobiomodulation (tPBM) followed by adjunctive swLORETA neurofeedback as part of routine clinical care. Clinical records and available standardized outcome measures were reviewed retrospectively after completion of the observation windows. No interventions were introduced for research purposes, and no prospective treatment protocol, randomization, blinding, or control condition was used.

Because management of PANS/PANDAS commonly involves concurrent immunological, gastrointestinal, metabolic, pharmacological, environmental, and behavioral interventions, this case series was not designed to isolate the effect of any single treatment component. The objective was descriptive: to document clinical, cognitive, functional, and qEEG observations during defined periods in which individualized neuromodulation formed part of broader multidisciplinary care. Findings are therefore exploratory and hypothesis-generating only.

### Participants

Two adolescent males with complex neuropsychiatric histories consistent with PANS/PANDAS were included. Both had histories of obsessive-compulsive symptoms or related neuropsychiatric symptoms, cognitive dysfunction, emotional dysregulation, gastrointestinal involvement, and functional impairment. Both had previously received extensive multidisciplinary care over more than 10 years, with incomplete resolution of symptoms.

The distinction between PANS and PANDAS was based on the working clinical diagnoses documented in the participants’ medical records. Both participants had histories consistent with streptococcal-associated symptom exacerbations and had been diagnosed with PANDAS at various points during their clinical course. However, both also experienced symptom exacerbations associated with non-streptococcal triggers and were subsequently managed within the broader PANS clinical framework. This retrospective report did not independently adjudicate diagnostic subtype or current diagnostic status. Accordingly, the term “PANS/PANDAS” is used throughout to reflect the participants’ documented clinical histories rather than to imply a single definitive diagnosis for either case.

Participant characteristics and intervention exposure are summarized in **Table 1**.

**Table 1:** Participant characteristics at baseline and intervention exposure.

Case	Age	Working clinical diagnosis	Duration of illness	Major interventions (before observation window)	tPBM protocol	Neurofeedback	Key functional impairments (baseline)
Case 1	14-year-old male	PANS/PANDAS, IBS-D	>10 years	GI, metabolic, immune/infectious, TruDOSE™ PRP†	1070 nm, 40 Hz → 10 Hz, 16 min 2x/day, 5–7 days/week†	13 sessions, swLORETA†	Severe executive dysfunction, attention deficits, emotional dysregulation, academic decline, IBS-D
Case 2	16-year-old male	PANS/PANDAS, ADHD, IBS-C	>10 years	GI, metabolic, immune/infectious, IVIG, sleep and attention medications‡	1070 nm, 40 Hz → 10 Hz, 16 min 2x/day, 5–7 days/week‡	41 sessions, swLORETA‡	Severe OCD, insomnia, homebound, anxiety, emotional dysregulation, academic decline, IBS-C

GI, gastrointestinal-directed therapy; metabolic, nutritional and methylation support; immune/infectious, therapies targeting immune dysregulation and chronic infection; PRP, platelet-rich plasma–based protocol; IVIG, intravenous immunoglobulin.

† Case 1: tPBM estimated at ~95 treatment days based on 5 days/week over 19 weeks. Neurofeedback was conducted over approximately 13 weeks. Major supportive interventions predated the observation window. GI-directed care, nutritional support, environmental management, a homeopathic *D. fragilis*-directed protocol, and acupuncture continued during follow-up. No major new systemic therapy was introduced during the observation window.

‡ Case 2: tPBM was estimated at ~225 treatment days based on 5 days/week over 45 weeks. Earlier self-directed home use occurred from January 2023 to July 2024 but was not formally monitored and could not be reliably quantified. Neurofeedback was conducted over approximately 44 weeks. Prior IVIG and attention medication predated the observation window. GI-directed care, nutritional support, environmental management, and sleep medication continued during follow-up. Quetiapine was discontinued approximately six weeks before the final assessment.

### Interventions

#### Transcranial photobiomodulation

Participants used a 1070 nm (± 50 nm) near-infrared tPBM device (Neuradiant 1070, Neuronic; 256 LEDs) at home as part of routine care. The typical schedule was 16 minutes twice daily, approximately 5–7 days per week. Initial stimulation parameters included 40 Hz pulsing at 75% intensity; in both cases stimulation was later changed to 10 Hz in response to tolerability concerns. For Case 1, this change occurred approximately 4 weeks after neurofeedback was introduced; for Case 2, approximately 12 weeks after. Because pulse frequency was not systematically manipulated, no conclusions can be drawn regarding frequency-specific effects.

Case 2 had an earlier period of self-directed home tPBM use from January 2023 through July 2024, before the formal observation window. This earlier use was not monitored prospectively and is presented as historical exposure only.

#### swLORETA neurofeedback

After an initial period of home tPBM, both participants underwent adjunctive weekly swLORETA neurofeedback in a clinical setting. Neurofeedback was delivered as routine clinical care by the treating neurofeedback clinicians. Case 1 completed 13 sessions over approximately 13 weeks, and Case 2 completed 41 sessions over approximately 44 weeks.

Protocols were individualized according to qEEG findings and clinical presentation and were modified over time based on clinician judgment, participant tolerability, and observed clinical response, with protocol progression determined by the treating clinician rather than by a predefined research protocol.

The author had no role in protocol selection, threshold setting, session delivery, or protocol modification. Complete session-level technical parameters, including exact target regions, reward and inhibit parameters, threshold settings, reinforcement parameters, and session-by-session protocol modifications, were not available retrospectively and therefore cannot be fully reported. This limits reproducibility and is acknowledged as a methodological limitation.

#### Concurrent treatments and clinical context

Both participants received multiple prior and concurrent supportive interventions outside the neuromodulation protocol, including gastrointestinal-directed care, metabolic and nutritional support, immune/infectious interventions, and environmental exposure management. In Case 2, intravenous immunoglobulin and attention medication predated the observation window, whereas sleep medication continued during follow-up. Major prior interventions are summarized in **Table 1**; interventions that continued during the observation window and the clinically relevant medication change, quetiapine discontinuation in Case 2, are described in the **Table 1** footnotes. Because of the retrospective design and concurrent interventions, the independent contribution of tPBM, neurofeedback, or their combined use cannot be determined; findings are presented descriptively.

### Outcome measures

Multimodal outcomes were selected from measures available in the clinical record and included symptom severity measured by the PANS-31 (10); obsessive-compulsive symptom severity by the Y-BOCS (16); cognitive performance using BrainGauge (Cortical Metrics LLC) (11, 12) and CogniFit CAB-Pro (CogniFit Inc.) (13–15); caregiver-reported functional outcomes; and qEEG interpreted by an independent qEEG consultant.

BrainGauge is a vibrotactile somatosensory device that assesses cortical function through fingertip stimulation across seven scored domains (speed, accuracy, temporal order judgment, time perception, plasticity, fatigue, and focus), combined into a composite Overall score (0–100) (11, 12). CogniFit CAB-Pro is a computerized battery comprising 17 tasks assessing 22 cognitive skills across five domains, with norm-referenced composite scores adjusted for age and sex (13–15). Both instruments were available within the clinical record and provided adjunctive measures of cortical and cognitive function; however, repeated testing may be influenced by practice effects, test familiarity, motivation, and day-to-day variability, and neither has been extensively validated in pediatric PANS/PANDAS populations. Findings from these assessments were interpreted as exploratory observations rather than definitive evidence of cognitive change.

Caregiver-reported functional outcomes were abstracted from clinical records and digital journal entries. Because they were not obtained under blinded conditions during active treatment, they may be influenced by expectancy effects and reporting bias.

#### Assessment timeline

**Figure 1:**
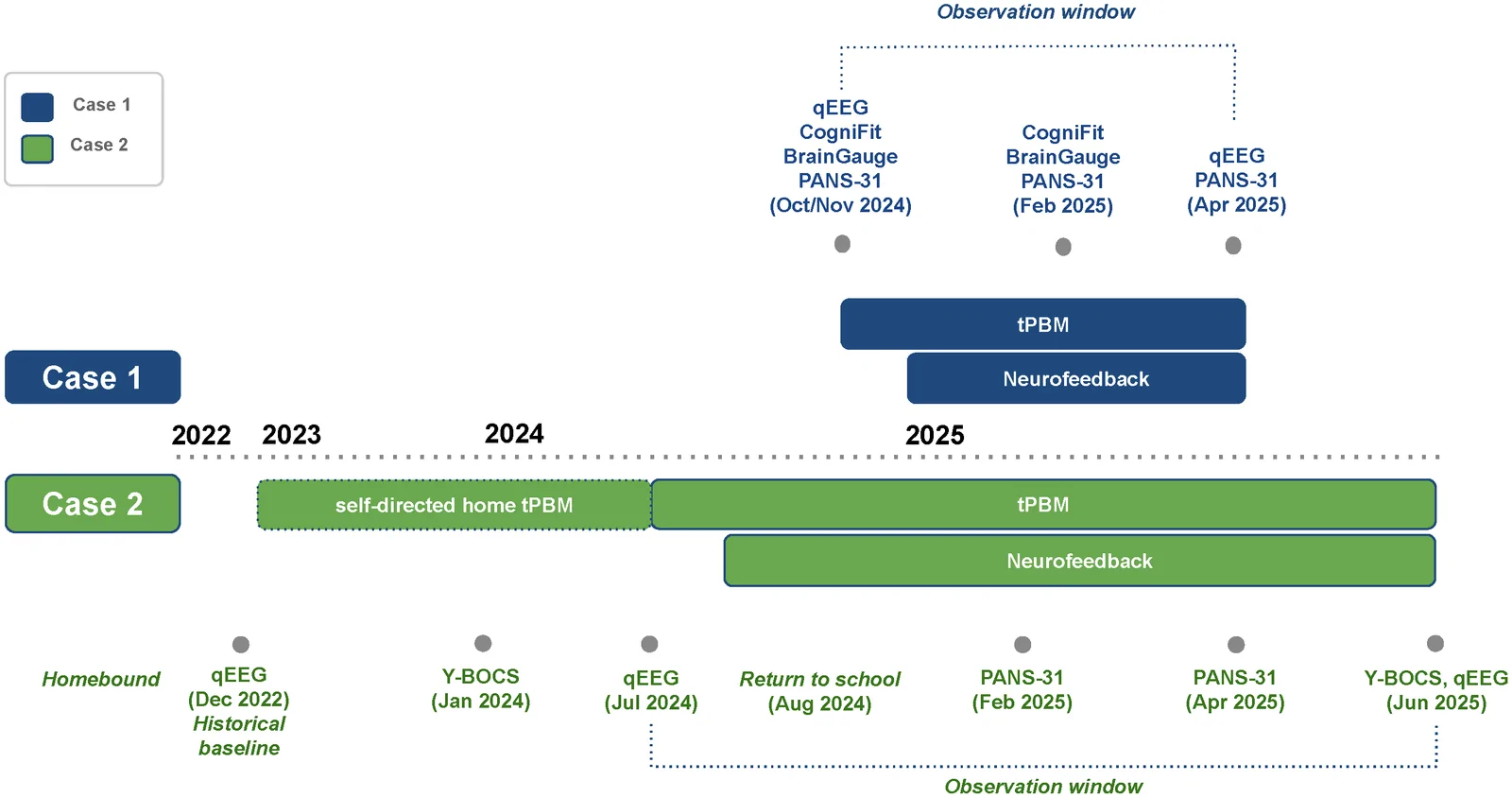
Timeline of intervention phases and outcome assessments for both cases. For Case 1, the formal observation window extended from late November 2024 to mid-April 2025 (19 weeks). For Case 2, the formal observation window extended from late July 2024 to early June 2025 (45 weeks). An earlier period of self-directed home tPBM use is shown separately for Case 2. A qEEG obtained in December 2022 is shown as a historical baseline providing longitudinal context prior to the observation window. swLORETA neurofeedback was introduced during the formal observation window. Outcome assessments were obtained at baseline and at selected follow-up timepoints.

Intervention phases and assessment timing are summarized in **Figure 1**. For Case 1, the formal observation window ran from November 30, 2024, to April 15, 2025 (19 weeks). Baseline assessments included qEEG (October 27, 2024), BrainGauge and CogniFit (November 2–3, 2024), and PANS-31 (November 3, 2024). A midpoint PANS-31 was obtained on February 20, 2025; post-intervention BrainGauge and CogniFit on February 24–25, 2025; and qEEG and PANS-31 follow-up on April 13 and 15, 2025.

For Case 2, the formal observation window ran from July 22, 2024, to June 3, 2025 (45 weeks). A Y-BOCS baseline was available from January 24, 2024, with follow-up on June 2, 2025. qEEG assessments were obtained on December 28, 2022 (historical baseline), July 22, 2024, and June 3, 2025. PANS-31 was introduced during the observation window and was not available as a true pre-intervention baseline; assessments were obtained on February 10 and April 19, 2025.

Because some measures preceded the observation window and others were introduced after treatment began, comparisons should be interpreted cautiously.

### qEEG acquisition and interpretation

qEEG recordings were obtained using a BrainMaster Discovery 20 system with Avatar software by an independent licensed clinician. Raw EEG data were subsequently reviewed by a separate independent qEEG consultant using NeuroGuide (Applied Neuroscience Inc.) with standardized normative comparisons. Recordings were processed using software-based artifact rejection and manual visual data pruning; artifacts were identified at the lead level and rejected where possible. When residual artifact could not be fully removed, this was noted in the written interpretation. Final reports were presented using a Laplacian montage for consistency across timepoints. Where reported, the theta-beta ratio was evaluated at Cz to minimize the influence of muscle artifact. Interpretation depth varied across timepoints because of differences in data quality.

## Results

### Overview

Two adolescents with persistent PANS/PANDAS symptoms received home-based transcranial photobiomodulation (tPBM) followed by adjunctive swLORETA neurofeedback as part of routine multidisciplinary care. Across the observation windows, changes were documented in symptom severity, cognitive performance, caregiver-reported functioning, and qEEG findings. Because assessment timing differed across participants and instruments, the findings are presented descriptively and should not be interpreted as evidence of treatment efficacy or treatment-specific effects.

### Case 1

At baseline, caregiver-reported symptoms included severe impairments in attention, sequencing, executive function, and emotional regulation, along with difficulties in procedural thinking and academic tasks. Prior interventions included functional medicine-based gastrointestinal treatment, metabolic and nutritional support, management of environmental exposures, and a platelet-based therapy protocol (TruDOSE™ PRP), without sustained resolution of neuropsychiatric symptoms. Available records indicated that major supportive interventions remained stable during the formal observation window.

Case 1 was followed over a 19-week observation window. Following an initial 4- to 5-week period of tPBM alone, BrainGauge and CogniFit assessments were obtained after approximately 7 weeks of combined tPBM and neurofeedback. PANS-31 was assessed at baseline, midpoint, and near the close of the observation window. qEEG was assessed at baseline and follow-up. These differences in assessment timing should be considered when interpreting findings across outcome measures.

**Figure 2:**
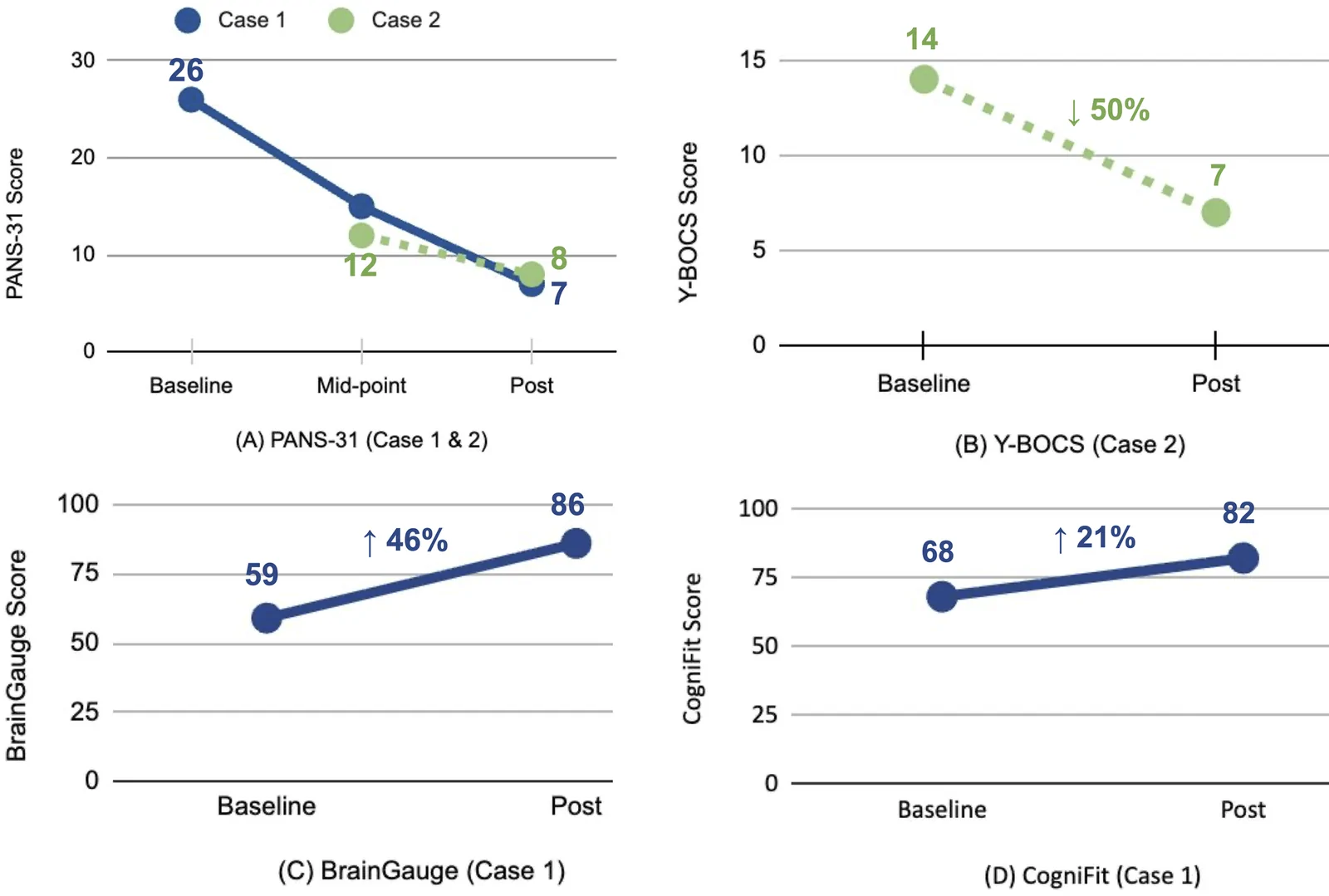
Clinical and cognitive outcomes across intervention phases. **(A)** PANS-31 (Cases 1 and 2). Case 1 demonstrated a progressive reduction from 26 to 15 to 7 (73% reduction). Case 2 scores were first available at midpoint (12), declining to 8 (33% reduction from first available assessment); a true baseline was not available. *PANS 31-Item Symptom Rating Scale (PANS Scale) ^©^ 2024 by Tanya K. Murphy, MD, MS, & Gail A. Bernstein, MD is licensed under CC BY-NC-SA 4.0.* **(B)** Y-BOCS score decreased from 14 to 7 in Case 2 (50% reduction). **(C)** BrainGauge performance increased from 59 to 86 in Case 1, representing a 46% increase. **(D)** CAB-Pro composite score increased from the 68th to the 82nd percentile in Case 1, representing an approximately 21% relative increase.

PANS-31 scores decreased from 26 at baseline to 15 at midpoint and 7 at follow-up, representing a 73% reduction from baseline (**Figure 2**). Item-level improvements were observed in attention-related symptoms, sleep disturbance, emotional regulation, and sensory amplification. Caregiver-reported functional changes during the same period included improved academic engagement, increased endurance, and greater self-motivation.

BrainGauge Overall score increased from 59 to 86 (**Figure 2**), with improvements across most measured cortical processing domains (**Supplementary Table 1**). The largest gains were observed in time perception (30 to 100), fatigue resistance (43 to 100), and accuracy (47 to 93). Temporal order judgment remained unchanged (53 to 53), and Focus showed a small decline (100 to 96), while remaining near the ceiling.

CogniFit CAB-Pro demonstrated improvement across 16 of 22 measured cognitive domains (**Supplementary Table 2**; **Supplementary Figure 2**). The largest increases were observed in auditory perception (37 to 88), contextual memory (49 to 86), and recognition (44 to 79). Executive-attention measures, including inhibition (+20) and updating (+16), also improved. Six domains showed small declines (−2 to −5 points), all remaining within or above average. Divided attention remained below average at both assessments (41 to 38). The composite score increased from 68 to 82 (**Figure 2**).

Caregiver reports described early variability following neurofeedback initiation, followed by stabilization and improvement over subsequent weeks. qEEG comparison showed mixed findings, with reductions in excess beta and high beta activity but persistent slowing in some posterior regions (**Supplementary Figure 1**). Interpretation remained limited by recording artifact and muscle tension; these findings are presented as exploratory physiological observations only.

### Tolerability and adverse events

In Case 1, transient paresthesia, described as a “pins and needles” sensation along the spine, was reported early during the tPBM course and diminished with continued use. No adverse events were reported in Case 2. In both cases, subjective overstimulation reported during 40 Hz pulsed tPBM was managed clinically by transitioning to 10 Hz stimulation. Because stimulation frequency was adjusted according to clinical judgment rather than systematically evaluated, tolerability observations are descriptive only.

### Case 2

At baseline, Case 2 presented with severe obsessive-compulsive symptoms, chronic insomnia, anxiety, emotional dysregulation, and functional impairment resulting in prolonged school absence. The clinical history also included sensitivity to environmental triggers, including mold exposure. Prior interventions included intravenous immunoglobulin, medications for sleep and attention, and comprehensive functional medicine approaches without complete symptom resolution.

Case 2 was followed over a 45-week formal observation window. Y-BOCS score declined from 14 to 7, representing a 50% reduction in obsessive-compulsive symptom severity (**Figure 2**). PANS-31 scores declined from 12 to 8 between the first available assessment and follow-up. However, the first PANS-31 assessment was obtained during the observation window and therefore does not represent a true pre-intervention baseline. Descriptive improvements were observed in domains including obsessions, attention, sleep, and emotional regulation.

Caregiver-reported functional changes included greater emotional flexibility, increased affectionate behavior, and improved daily functioning. Functionally, the participant progressed from prolonged homebound status to full school participation, including academic engagement and extracurricular involvement, while maintaining high academic performance.

**Figure 3:**
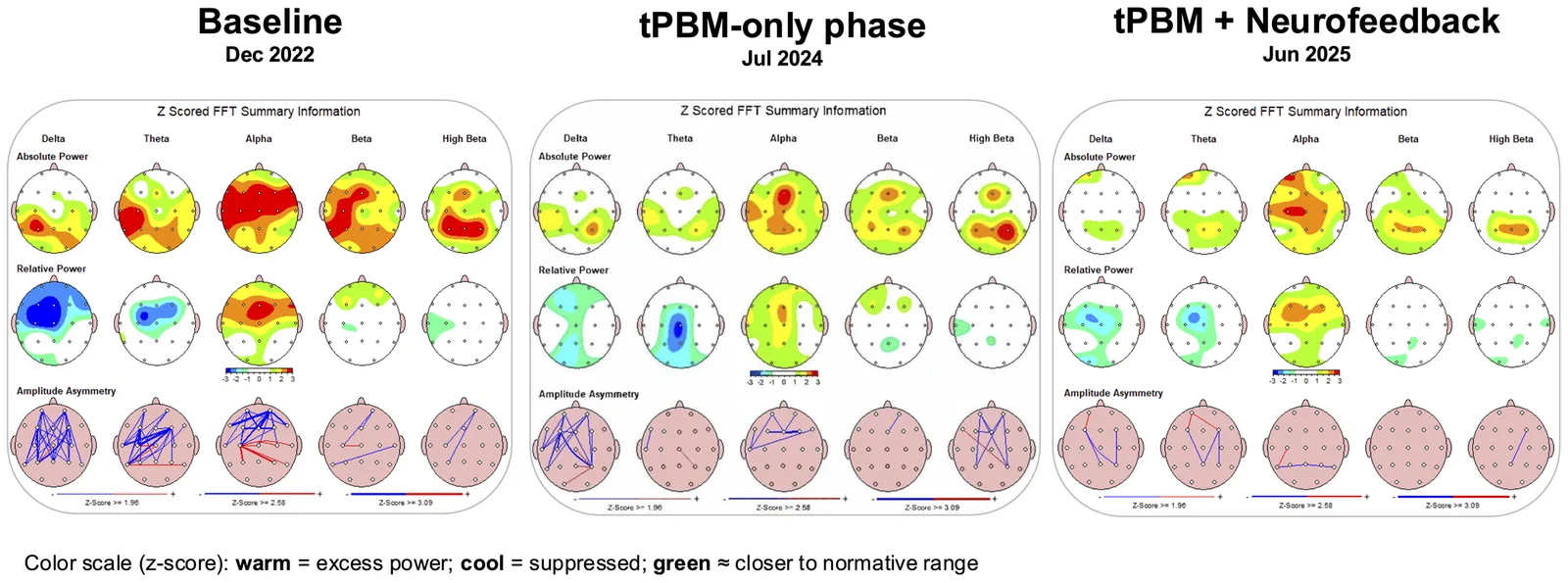
Sequential qEEG maps across intervention phases in Case 2. Three assessment timepoints are shown: a historical baseline in December 2022, before the formal observation window; a tPBM-only phase in July 2024, at the onset of the formal observation window; and a combined tPBM plus neurofeedback phase in June 2025, at the close of the observation window. Each panel displays z-scored FFT summary maps across three rows representing absolute power, relative power, and amplitude asymmetry, and across five frequency bands (delta, theta, alpha, beta, and high beta). Values are referenced against age-matched normative data. Warm colors indicate excess power relative to age-matched normative data, cool colors indicate suppressed power, and green generally reflects values closer to the normative range. Maps were generated using NeuroGuide (Applied Neuroscience Inc.), processed using a Laplacian montage, and reviewed by an independent qEEG consultant. Descriptively, patterns of dysregulation appeared to become more localized across timepoints, with some z-scores moving closer to normative ranges and relative power distribution appearing more organized at the final assessment. Posterior dysregulation was reduced but not fully normalized. The theta-beta ratio measured at Cz in the eyes-open state remained substantially below the age-based normative range at the documented assessments, consistent with persistent hyperarousal as reported by the interpreting consultant. These maps are presented for descriptive comparison only and should not be interpreted as evidence of treatment efficacy or neurophysiological normalization.

In Case 2, qEEG findings across three assessments showed longitudinal changes spanning a historical baseline, the tPBM-only phase, and the combined tPBM plus neurofeedback phase (**Figure 3**). Descriptively, patterns of dysregulation appeared to become more localized over time, with some z-scores moving toward normative ranges and relative power distribution appearing more organized across assessments. Posterior dysregulation was reduced but not fully normalized, with abnormalities appearing more confined to posterior regions than at the historical baseline. The theta-beta ratio at Cz in the eyes-open state remained substantially below the age-based normative range at the documented assessments, consistent with persistent hyperarousal as reported by the interpreting consultant. These qEEG findings are presented as exploratory physiological observations and should not be interpreted as evidence of a specific treatment mechanism.

Quetiapine, used for sleep support, was discontinued approximately six weeks before the final assessment. Clinical improvements were observed before discontinuation and were maintained afterward; however, the contribution of medication changes or other concurrent clinical factors cannot be determined.

Pre-study clinical data provide additional longitudinal context but were not collected under research conditions. Historical NeuroQuant^®^, BrainGauge, and qEEG findings documented severe prior abnormalities during periods of peak symptom burden. These findings are presented only as clinical context and should not be interpreted as study outcomes.

### Cross-case patterns

Across both cases, reductions in neuropsychiatric symptom burden, improvements in caregiver-reported attention and emotional regulation, and functional gains in academic engagement and daily activities were observed across multiple outcome domains, including standardized symptom ratings, cognitive assessments, and qEEG findings, during periods when tPBM and neurofeedback were incorporated into broader multidisciplinary care. Improved tolerability was noted following transition from 40 Hz to 10 Hz tPBM in both cases. Persistent vulnerabilities remained, including unchanged temporal order judgment and below-average divided attention in Case 1 and residual qEEG abnormalities in Case 2.

These findings should be interpreted cautiously. The two participants differed in baseline severity, duration of observation, prior tPBM exposure, concurrent treatments, and assessment schedules. Because of the retrospective design, very small sample size, and multiple potential confounding factors, no conclusions can be drawn regarding the independent or combined effects of tPBM and neurofeedback.

## Discussion

This retrospective case series provides descriptive observations across multiple clinical, cognitive, functional, and neurophysiological domains in two adolescents with persistent PANS/PANDAS who received individualized tPBM followed by adjunctive swLORETA neurofeedback as components of broader multidisciplinary care. The findings do not establish that tPBM, neurofeedback, or their combination caused the observed improvements. Both participants had complex treatment histories involving immunological, gastrointestinal, metabolic, pharmacological, environmental, and behavioral interventions, any of which may have contributed to clinical change, and Case 2 had prolonged self-directed tPBM exposure before the formal observation window. These factors limit attribution; findings should be interpreted descriptively, not as evidence of efficacy.

Despite these limitations, the convergence of observations across multiple outcome domains may warrant further study. Both participants showed improvement in standardized symptom measures and caregiver-reported functioning during periods when neuromodulation was incorporated into multidisciplinary care. Case 1 also demonstrated improvements across several BrainGauge and CogniFit domains, whereas Case 2 progressed from prolonged homebound status to full school participation. These observations may help identify outcome domains and patient characteristics that merit prospective evaluation.

Notably, several domains demonstrating the largest changes, including memory, auditory perception, and executive function, are among those commonly affected in PANS/PANDAS (1, 2).

One hypothesis generated by these cases is that adjunctive neuromodulation may have a role in selected patients with persistent neuropsychiatric or functional symptoms despite extensive systemic care. This is compatible with current models of PANS/PANDAS involving immune-mediated disruption of cortico-striatal and related regulatory circuits (1, 3). However, the present report did not directly evaluate underlying biological mechanisms, and mechanistic interpretations remain speculative.

The temporal sequencing of tPBM followed by neurofeedback also generates hypotheses for future study. In both cases, some clinical changes were observed during the tPBM phase, with additional changes documented after neurofeedback was introduced. Because treatment phases were neither randomized nor prospectively standardized, these observations cannot establish additive or synergistic effects. One proposed framework is that tPBM may influence physiological conditions relevant to cortical function (4–6), whereas neurofeedback may provide repeated training of neural self-regulation (7–9). Whether these modalities produce independent, additive, or synergistic effects remains unknown and should be evaluated in prospective studies comparing tPBM alone, neurofeedback alone, combined treatment, and appropriate comparison groups.

qEEG findings provided exploratory neurophysiological context and should not be interpreted as definitive evidence of treatment response or mechanism. In Case 2, patterns of dysregulation appeared to become more localized over time, with some z-scores moving toward normative ranges; however, posterior abnormalities persisted and the theta-beta ratio at Cz remained substantially below the age-based normative range at the documented assessments. In Case 1, reductions in excess beta and high beta activity were observed, though interpretation remained limited by recording artifact. Given the ongoing scientific debate regarding qEEG clinical utility, these findings should be regarded as adjunctive physiological observations only.

Case 1 demonstrated improvements across several BrainGauge and CogniFit measures, with the largest gains in time perception, fatigue resistance, auditory perception, and memory domains. However, repeated testing may be influenced by practice effects, motivation, fatigue, and day-to-day variability, and these tools have not been extensively validated in pediatric PANS/PANDAS populations. Persistent vulnerabilities, including unchanged temporal order judgment and continued below-average divided attention, indicate that improvements were incomplete and domain specific.

The persistence of temporal order judgment impairment in Case 1, together with historical BrainGauge abnormalities and residual qEEG findings in Case 2, raises the hypothesis that some patients with PANS/PANDAS may retain measurable network-level vulnerabilities despite improvement in conventional symptom measures. Temporal order judgment has been associated with cortical timing and integration processes involving distributed frontal, parietal, and sensory networks (11, 20, 21). Future prospective studies using standardized cognitive batteries, repeated qEEG acquisition, and longitudinal follow-up are needed to determine whether these measures track illness activity, recovery, or persistent impairment.

Both participants had longstanding gastrointestinal and systemic medical histories. Although the present study was not designed to evaluate gut-brain mechanisms, emerging literature has suggested bidirectional interactions between gastrointestinal, immune, and neurobehavioral systems (22). Whether such factors influence responsiveness to neuromodulation in PANS/PANDAS remains unknown and warrants future investigation.

Tolerability observations remain preliminary. Both participants initially used 40 Hz pulsed tPBM and later transitioned to 10 Hz following reports of overstimulation, with improved tolerability subsequently reported. Because stimulation frequency was adjusted clinically during overlapping neurofeedback periods, it is not possible to determine whether tolerability differences reflected tPBM, neurofeedback, their interaction, or other concurrent factors. The initial use of 40 Hz was informed by literature suggesting potential relevance of gamma-frequency stimulation to attention and higher-order information processing (17). The transition to 10 Hz was guided primarily by tolerability. Lower-frequency pulsed protocols have been described in the tPBM literature (18), and alpha-frequency activity (approximately 8–12 Hz) has been associated with reduced arousal and cortical inhibition (19). Although the relevance of these frequency-related concepts to the present observations remains speculative, the differing tolerability observed across frequencies may warrant prospective investigation.

## Limitations

Several limitations must be acknowledged when interpreting these findings. First, this study represents a retrospective case series of only two participants without a control group, randomization, blinding, or a prospectively defined treatment protocol. Both participants received multiple prior and concurrent interventions, including immunological, gastrointestinal, metabolic, nutritional, environmental, behavioral, and pharmacological therapies, and the relative contributions of individual interventions, either neuromodulation modality, or their combined use cannot be determined. In addition, Case 2 had approximately 18 months of self-directed home tPBM use before the formal observation window, making it impossible to distinguish potential cumulative effects of prior exposure from observations made during the study period.

Standardized assessments were not obtained uniformly across participants or timepoints. Some baseline measures were collected before the formal observation window, whereas others, including the PANS-31 in Case 2, were introduced after treatment had already begun. Furthermore, caregiver-reported functional outcomes were not collected under blinded conditions and may have been influenced by expectancy effects, observer bias, placebo effects, regression to the mean, spontaneous symptom fluctuation, maturation, or the natural course of illness.

The neurofeedback intervention was individualized according to each participant’s qEEG-identified patterns of dysregulation and clinical presentation and delivered as routine clinical care. Because treatment was not conducted under a prospective research protocol, complete session-level details, including exact target regions, reward and inhibit parameters, threshold settings, reinforcement parameters, session progression, and protocol modifications, were not available retrospectively. This limits reproducibility and the ability to independently evaluate or replicate the intervention.

The qEEG findings should be interpreted cautiously. Although data were acquired and reviewed by independent clinicians, interpretation of the post-intervention assessment in Case 1 remained limited by recording artifact and muscle tension despite data pruning and artifact rejection procedures. Raw data were not subjected to blinded secondary interpretation or independent statistical reanalysis. qEEG interpretation remains an area of ongoing scientific discussion and should be viewed as exploratory rather than definitive evidence of treatment response or mechanism.

The cognitive outcome measures have important limitations. Repeated testing may be influenced by practice effects, test familiarity, motivation, fatigue, and day-to-day variability. The CAB-Pro composite score is derived from a proprietary algorithm not publicly available for independent verification, and neither BrainGauge nor CAB-Pro has been extensively validated in pediatric PANS/PANDAS populations.

Finally, selected historical clinical data, including NeuroQuant^®^ findings and earlier BrainGauge assessments, are presented as historical context and should not be interpreted as study outcomes or indicators of treatment response.

## Conclusion

This retrospective case series describes two adolescents with persistent PANS/PANDAS symptoms who showed improvements across clinical, cognitive, functional, and neurophysiological outcome measures while individualized tPBM and swLORETA neurofeedback were incorporated into broader multidisciplinary care. Because of the retrospective design, very small sample size, prolonged prior treatment histories, multiple concurrent interventions, non-uniform assessment schedules, and the absence of a control group, these observations should not be interpreted as evidence of efficacy or causal treatment effects.

The convergence of findings across clinical, cognitive, functional, and neurophysiological domains may represent an observational clinical signal that warrants further investigation and may generate testable hypotheses regarding adjunctive neuromodulation in PANS/PANDAS.

Future prospective studies should evaluate tPBM alone, neurofeedback alone, and their combined use using standardized intervention protocols, clearly documented concurrent therapies, blinded or independent outcome assessment, predefined assessment schedules, and appropriate comparison groups. Such studies are needed to determine whether these observations reflect reproducible therapeutic effects or other explanations.

## Data Availability

The original contributions presented in the study are included in the article/**Supplementary Material**. Further inquiries can be directed to the corresponding author.
