# Supplementary material for: Combined transcranial photobiomodulation and neurofeedback in PANS/PANDAS: a retrospective case series
Source: Front Psychiatry. 2026 Sep 3;17:1865423. doi: 10.3389/fpsyt.2026.1865423 (PMC13582508; doi:10.3389/fpsyt.2026.1865423)
Supplement: Supplementary file 1 [file Supplementaryfile1.docx]

**Supplementary Table S1.** Pre- and post-intervention Brain Gauge cortical metric scores — Case 1.

| **Domain** | **BrainGauge Pre** | **BrainGauge Post** | **Change** |
| --- | --- | --- | --- |
| **Time Perception** | 30 | 100 | +70 |
| **Fatigue** | 43 | 100 | +57 |
| **Accuracy** | 47 | 93 | +46 |
| **Plasticity** | 57 | 86 | +29 |
| **Speed** | 64 | 77 | +13 |
| **Temporal Order Judgment** | 53 | 53 | 0 |
| **Focus** | 100 | 96 | −4 |
| **Overall**^*^ | **59** | **86** | **+27** |

^*^*Scores are norm-referenced composite indices (0–100); higher scores indicate more optimal cortical processing relative to normative values. Pre-intervention assessment: November 2, 2024; post-intervention assessment: February 24, 2025.*

**Supplementary Table S2.** Pre- and post-intervention CogniFit CAB-Pro domain scores — Case 1.

| **Domain** | **CogniFit Pre** | **CogniFit Post** | **Change** |
| --- | --- | --- | --- |
| Auditory Perception | 37 | 88 | +51 |
| Contextual Memory | 49 | 86 | +37 |
| Recognition | 44 | 79 | +35 |
| Non-Verbal Memory | 68 | 99 | +31 |
| Visual Short-Term Memory | 68 | 98 | +30 |
| Working Memory | 61 | 90 | +29 |
| Planning | 72 | 97 | +25 |
| Inhibition | 63 | 83 | +20 |
| Visual Perception | 50 | 70 | +20 |
| Short-Term Memory | 79 | 97 | +18 |
| Hand-Eye Coordination | 39 | 57 | +18 |
| Updating | 76 | 92 | +16 |
| Naming | 71 | 87 | +16 |
| Shifting | 65 | 73 | +8 |
| Estimation | 74 | 77 | +3 |
| Response Time | 96 | 97 | +1 |
| Processing Speed | 92 | 90 | −2 |
| Visual Scanning | 81 | 79 | −2 |
| Focused Attention | 74 | 71 | −3 |
| Divided Attention | 41 | 38 | −3 |
| Spatial Perception | 88 | 84 | −4 |
| Phonological STM | 87 | 82 | −5 |
| **CAB-Pro Composite**^*^ | **68** | **82** | **+14** |

^*^*Scores are norm-referenced percentile indices (0–100); a score of 50 represents the population mean for age and sex. Domains listed in descending order of change. Five CogniFit domain composites (Memory, Attention, Reasoning, Coordination, Perception) are excluded as they are derived from the individual domains above. Pre-intervention assessment: November 3, 2024; post-intervention assessment: February 25, 2025.*

**Supplementary Figure S1.** Pre- and post-intervention qEEG findings in Case 1.

qEEG maps demonstrate baseline and post-intervention findings following a shorter intervention interval of combined tPBM and neurofeedback in Case 1. Reductions in excess beta and high beta activity are visible in relative power maps at post-intervention. Patterns of slowing in posterior temporal and parietal regions appeared more prominent in some areas at follow-up than at baseline, consistent with ongoing neurophysiological processing following a brief intervention course. Interpretation of the post-intervention assessment was limited in part by recording artifact and muscle tension. qEEG maps shown are eyes-closed recordings processed using a Laplacian montage. Data were acquired using a BrainMaster Discovery 20 amplifier with Avatar software, analyzed using NeuroGuide (Applied Neuroscience Inc.), and reviewed by an independent qEEG consultant. Maps represent z-scored FFT summary information across frequency bands.

**Supplementary Figure S2.** Change in CogniFit CAB-Pro domain scores from pre- to post-intervention — Case 1.

Horizontal bars represent the magnitude of change in each cognitive domain between pre-intervention (03 November 2024) and post-intervention (25 February 2025) assessments. Bars extending to the right indicate improvement; bars extending to the left indicate decline. Domains are ordered from largest to smallest improvement. Of 22 domains, 16 showed improvement and 6 showed minimal decline. Scores are norm-referenced percentile indices (0–100) adjusted for age and sex; a score of 50 represents the population mean. Full pre- and post-intervention scores for all domains are provided in Supplementary Table S2.
